# Enhanced Ultraviolet Damage Resistance in Magnesium Doped Lithium Niobate Crystals through Zirconium Co-Doping

**DOI:** 10.3390/ma14041017

**Published:** 2021-02-21

**Authors:** Tengfei Kong, Yi Luo, Weiwei Wang, Hanxiao Kong, Zhiqin Fan, Hongde Liu

**Affiliations:** 1 School of Sciences, Henan University of Technology, Zhengzhou 450001, China; kongtf@haut.edu.cn; 2 Key Laboratory for Special Functional Materials of Ministry of Education, Henan University, Kaifeng 475004, China; luoy61@163.com; 3 Department of Mathematics and Physics, Shijiazhuang Tiedao University, Shijiazhuang 050043, China; weiweiwang@mail.nankai.edu.cn; 4 College of Chemistry and Environmental Science, Hebei University, Baoding 071002, China; konghxiao@163.com; 5 MOE Key Laboratory of Weak-Light Nonlinear Photonics and School of Physics, Nankai University, Tianjin 300071, China; 6 TEDA Institute of Applied Physics, Nankai University, Tianjin 300071, China

**Keywords:** lithium niobate, ultraviolet damage resistance, defects, first-principles calculations

## Abstract

MgO-doped LiNbO_3_ (LN:Mg) is famous for its high resistance to optical damage, but this phenomenon only occurs in visible and infrared regions, and its photorefraction is not decreased but enhanced in ultraviolet region. Here we investigated a series of ZrO_2_ co-doped LN:Mg (LN:Mg,Zr) regarding their ultraviolet photorefractive properties. The optical damage resistance experiment indicated that the resistance against ultraviolet damage of LN:Mg was significantly enhanced with increased ZrO_2_ doping concentration. Moreover, first-principles calculations manifested that the enhancement of ultraviolet damage resistance for LN:Mg,Zr was mainly determined by both the increased band gap and the reduced ultraviolet photorefractive center O^2−/−^. So, LN:Mg,Zr crystals would become an excellent candidate for ultraviolet nonlinear optical material.

## 1. Introduction

Lithium niobate (LiNbO_3_, LN), also known as the silicon of nonlinear optics, is famous for its excellent physical properties such as acousto-optic, electro-optic, pyro-electric, piezoelectric, and nonlinear properties [1,2]. Although it has been grown for more than half a century, LiNbO_3_ has remained one of the hot issues for researchers up to now [3]. In LiNbO_3_, the optically-induced damage, also called the photorefraction, can be produced easily with a laser beam of several milliwatts, which seriously hinders its applications such as optical waveguides, frequency convertors, and Q-switches at high light intensities [4]. The good news is that doping MgO into LiNbO_3_ solves this problem well, and the optical damage resistance for LiNbO_3_ can be increased by two orders of magnitude, substantially promoting its practical use in nonlinear optics [5,6]. Nowadays, MgO doped LN (LN:Mg) still garners extensive research interest in optical waveguide, domain engineering, and quasi-phase-matching, however, it should be noted that most of these researches have only been carried out in visible and infrared regions, but not in ultraviolet (UV) [7,8]. In fact, LN:Mg exposes the visible enhancement of UV photorefraction [9], and this is a serious problem that has become a noteworthy impediment to its extension of applications into UV region.

As we know, the chemical element Zr is considered as a unique and promising dopant in LiNbO_3_ since it has a lower doping threshold (~ 2.0 mol.%) and a optimal distribution coefficient close to one, especially the strong optical damage resistance from UV to visible [10,11,12,13]. It should be pointed out that it has been the only doped LN crystal possessing superior resistance against UV damage so far [14]. Generally, this property is thought to be related to the replacement of Zr^4+^ ions against Nb^5+^ on Li sites because the element Zr is adjacent to Nb in the periodic table. It has been reported that Zr^4+^ ions co-dope with some transition metal elements in LN crystals [15,16], such as Fe, Mn, Cu, and Ce, which can considerably reduce the light-induced scattering as well as improve the response speed. Besides, Zr doping also improves the near infrared emission efficiency in LN:Er, LN:Yb,Er crystals [17]; meanwhile, it can also stabilize the signal output of the Ti-diffused LN waveguides under the high-power pumping without optical damage observed [18]. Furthermore, we have reported that some additional ZrO_2_ doping into LN:Mg not only can finely adjust the phase-matching temperature but also realize the room temperature noncritical phase-matching [19]. The above research results suggest that Zr doping have such a strong impact on the properties of LN, so further research on its influence on the UV photorefraction of LN:Mg is very necessary and meaningful to promote the laser frequency conversion to UV wavelength region. Therefore, in this work we mainly investigated the resistance against UV damage of ZrO_2_ co-doped LN:Mg crystals. Moreover, the first-principles calculations were employed to explore the effect of the crystal defect on the UV photorefractive properties in the doubly-doped LiNbO_3_.

## 2. Materials and Methods

A series of fixed 5.0 mol.% MgO and various ZrO_2_ (0, 0.5, 1.0 and 1.5 mol.%) co-doped LiNbO_3_ crystals were investigated and labelled as LN:Mg_5.0_,Zr_0.5_, LN:Mg_5.0_,Zr_1.0_, and LN:Mg_5.0_,Zr_1.5_, respectively. For comparison, the 2.0 mol.% ZrO_2_-doped congruent LN crystal was also prepared, labeled as LN:Zr_2.0_. Here, the crystal growth and treatment had been described in our previous report [19].

The transmitted beam spot distortion method [20] was performed directly and evaluated the optical damage resistance of crystals in the UV region. An *e*-polarized beam operating at 355 nm from a continuous wave frequency-tripled solid-state laser with a maximum output power of 100 mW was focused into the 3.0-mm-thick *y*-plates. The laser beam was polarized parallel to the *c*-axis of the plates, and the highest intensity at the focal point was 2.2 × 10^5^ W/cm^2^. When a crystal suffers from the optical damage, the transmitted beam spot is smeared and elongated along the *c*-axis with decreased intensities at the central part. Moreover, in order to further characterize the optical damage resistance at 355 nm quantitatively, we also used the two-wave coupling holographic recording method [21], namely, two coherent *e*-polarized beams with irradiation intensities of 400 mW/cm^2^ were focused into the *y*-oriented plates at the intersecting angle of 28° (2*θ*) to measure the light-induced refractive index changes (Δ*n*) of these crystals. 

In addition, to explore the linking mechanism between defect structure and optical damage resistance properties, the theoretical calculation for the defects and electronic structure of the crystals was performed by the Vienna Ab-initio Simulation Package (VASP) and general gradient approximation (GGA) [22,23]. The cutoff energy of the projector augmentation wave (PAW) pseudo-potentials was 400 eV with an allowed error in energy from relaxation of 10^−5^ eV. Moreover, the calculations of the bulk defects in perfect LN were carried out in the super-cell containing 2 × 2 × 1 conventional unit cells with 120 atoms, meanwhile the employed 2 × 2 × 1 *K*-points mesh over the Brillouin-zone was generated by the Monkhost–Pack scheme [24].

## 3. Results and Discussion

### 3.1. Resistance Against Ultraviolet Damage

**Figure 1:**
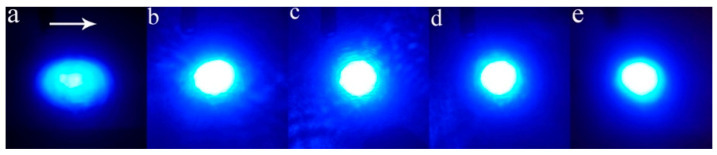
Distortion of transmitted laser beam spots after 5 min of irradiation. The white arrow represents the *c*-axis of the crystal. (**a**) LN:Mg_5.0_; (**b**) LN:Mg_5.0_,Zr_0.5_; (**c**) LN:Mg_5.0_,Zr_1.0_; (**d**) LN:Mg_5.0_,Zr_1.5_; (**e**) LN:Zr_2.0_. The light intensities are (**a**) 2.8 × 10^3^ W/cm^2^ and (**b**–**e**) 2.2 × 10^5^ W/cm^2^.

Figure 1 depicts the images of the transmitted beam spots for different samples after 5 min radiation. With the ZrO_2_ doping concentration increasing from 0.5 to 1.5 mol.%, no beam spot distortion is observed in LN:Mg,Zr crystals with a maximum light intensity of 2.2 × 10^5^ W/cm^2^, as in the case of LN:Zr_2.0_. For comparison, the transmitted light beam spot of LN:Mg_5.0_ obviously diffuses along the *c*-axis under the low light intensity of 2.8 × 10^3^ W/cm^2^. Based on the results above, we found out that the resistance against UV optical damage of LN:Mg_5.0_ is nearly improved by two orders of magnitude by only 0.5 mol.% ZrO_2_ doping.

**Figure 2:**
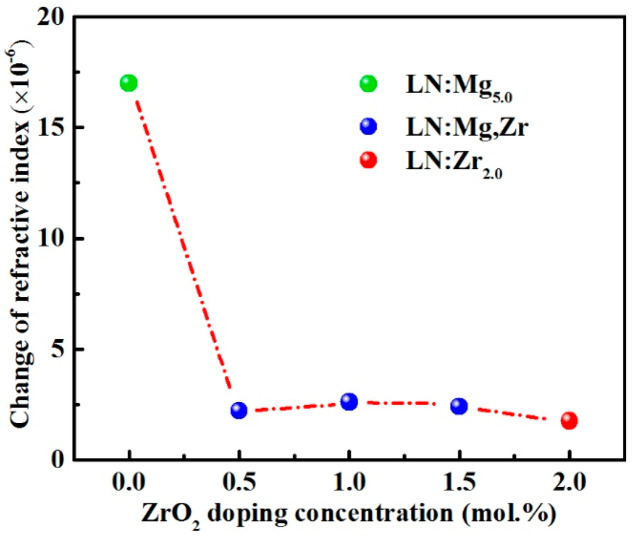
Refractive index changes of LN:Mg,Zr crystals as a function of ZrO_2_ doping concentration. That of LN:Zr_2.0_ is labelled for comparison.

We also calculated the refractive index changes by two-wave coupling holographic recording method, and the relevant results are shown in Figure 2. From this figure, it reveals that the changes of the refractive index of LN:Mg_5.0_ have lowered about an order of magnitude by co-doping with ZrO_2_, which can even be compared with that of LN:Zr_2.0_. Considering the inverse correlation in the change of refractive index with the optical damage resistance of the crystals, we conclude that the additional ZrO_2_ dopants greatly reduce the UV optical damage of LN:Mg_5.0_. However, it does not show a linear pattern between the refractive index changes and ZrO_2_ co-doping with increasing ZrO_2_ doping concentration in doubly crystals, and this particularity probably signifies that ZrO_2_ doping has had a profound effect on the crystal structure of LN:Mg. So, we utilized the first-principles calculations to find out more about the inherent mechanism of enhanced UV optical damage resistance of LN:Mg by ZrO_2_ co-doping.

### 3.2. Defects Structure in the LN:Mg,Zr

**Figure 3:**
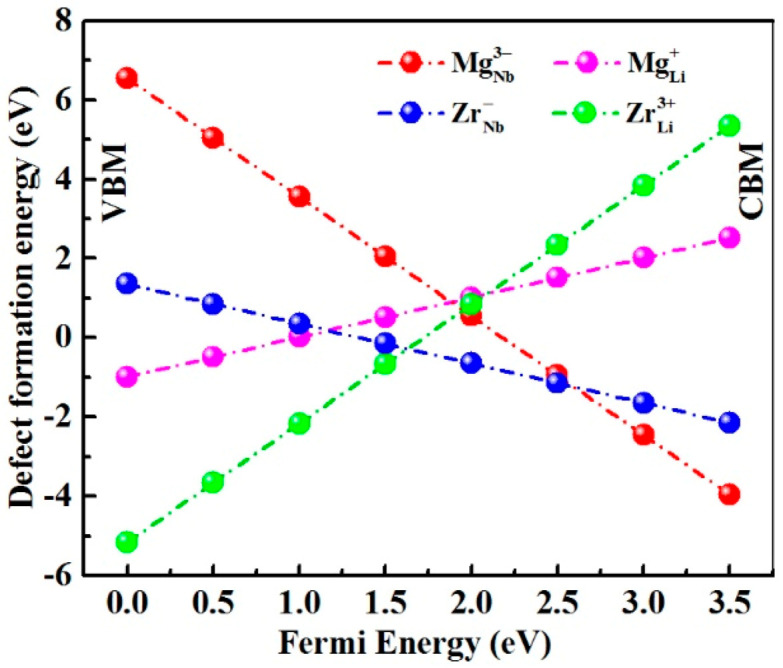
Formation energies of point defects ${\mathrm{Mg}}_{\mathrm{Li}}^{+}$, ${\mathrm{Mg}}_{\mathrm{Nb}}^{3-}$,${\;\mathrm{Zr}}_{\mathrm{Li}}^{3+}$,${\;\mathrm{Zr}}_{\mathrm{Nb}}^{-}$ in LN:Mg,Zr crystals. The valance-band maximum and the conduction-band minimum are labelled as VBM and CBM, respectively. Fermi energy (E_F_) range corresponds to the fundamental band gap of LN.

Generally, the formation energy is treated as the stability criterion of defects in materials [25]. According to the optimization model of prefect LN [26], we firstly calculated the formation energies of point defects ${\;\mathrm{Mg}}_{\mathrm{Li}}^{+}$, ${\mathrm{Mg}}_{\mathrm{Nb}}^{3-}$,${\;\mathrm{Zr}}_{\mathrm{Li}}^{3+}$, ${\;\mathrm{Zr}}_{\mathrm{Nb}}^{-}\;$ in LN:Mg,Zr to investigate the substitution sequence of doping ions, and the results are shown in Figure 3. It should be noted that the concentration increase of doping ions will cause the raise of Fermi level [27], so it can be observed from the figure that ${\mathrm{Mg}}_{\mathrm{Li}}^{+}$ and ${\mathrm{Zr}}_{\mathrm{Li}}^{3+}$ transform into ${\mathrm{Mg}}_{\mathrm{Nb}}^{3-}$ and ${\mathrm{Zr}}_{\mathrm{Nb}}^{-}$ at the points when E_F_ = 1.9 eV and E_F_ = 1.6 eV, respectively, which illustrates that Mg^2+^ and Zr^4+^ ions give priority to form ${\mathrm{Mg}}_{\mathrm{Li}}^{+}$ and ${\mathrm{Zr}}_{\mathrm{Li}}^{3+}$ rather than ${\mathrm{Mg}}_{\mathrm{Nb}}^{3-}$ and ${\mathrm{Zr}}_{\mathrm{Nb}}^{-}$. It is widely accepted [14] that doping ions in LN are assumed preferably to occupy Li sites and replace anti-site ${\mathrm{Nb}}_{\mathrm{Li}}^{4+}$ up to the threshold value, and further above the threshold value, all ${\mathrm{Nb}}_{\mathrm{Li}}^{4+}$ defects are replaced, then doping ions separately enter Nb sites and Li sites. Clearly, Zr^4+^ ions occupy Nb sites ahead of Mg^2+^ ions.

In our previous report [19] we employed the OH^–^ absorption spectra and UV-visible absorption spectra to analyze the defects of these doubly co-doped crystals, and the results indicated that the ${\mathrm{Mg}}_{\mathrm{Li}}^{+}$ + ${\mathrm{Zr}}_{\mathrm{Nb}}^{-}$ defect clusters exist in crystals. Hence, according to the experimental results and the above calculated results of point defects, three kinds of defect clusters were constructed to find the energetically preferable configuration in LN:Mg,Zr crystals:

1. Zr^4+^ and Mg^2+^ ions only occupy Li-sites, labelled as ${\mathrm{Zr}}_{\mathrm{Li}}^{3+}$ + ${4V}_{\mathrm{Li}}^{-}$ + ${\mathrm{Mg}}_{\mathrm{Li}}^{+}$;
2. Zr^4+^ and Mg^2+^ ions replace Nb- and Li-sites, respectively, labelled as ${\mathrm{Mg}}_{\mathrm{Li}}^{+}$ + ${\mathrm{Zr}}_{\mathrm{Nb}}^{-}$;
3. Zr^4+^ ions only substitute Nb-sites while Mg^2+^ ions occupy both Li- and Nb-sites, labelled as ${\mathrm{Zr}}_{\mathrm{Nb}}^{-}$ + ${4\mathrm{Mg}}_{\mathrm{Li}}^{+}$ + ${\mathrm{Mg}}_{\mathrm{Nb}}^{3-}$.

Table 1 shows the calculated defect formation energies, and the ${\mathrm{Mg}}_{\mathrm{Li}}^{+}$ + ${\mathrm{Zr}}_{\mathrm{Nb}}^{-}$ defect cluster had the lowest defect formation energy, reflecting that the existence of ${\mathrm{Mg}}_{\mathrm{Li}}^{+}$ + ${\mathrm{Zr}}_{\mathrm{Nb}}^{-}$ defect cluster in LN:Mg,Zr was reasonable, which is in agreement with that of our previous experimental results. 

### 3.3. Charge States of LN:Mg,Zr

**Figure 4:**
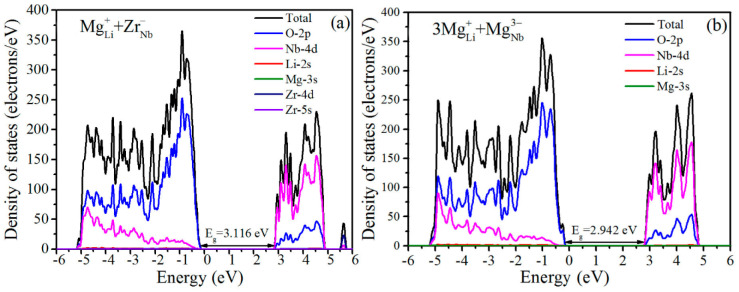
Density of states for (**a**) LN:Mg,Zr based on the ${\mathrm{Mg}}_{\mathrm{Li}}^{+}$ + ${\mathrm{Zr}}_{\mathrm{Nb}}^{-}$ defect model. For comparison, that of (**b**) LN:Mg based on the 3${\mathrm{Mg}}_{\mathrm{Li}}^{+}$ + ${\mathrm{Mg}}_{\mathrm{Nb}}^{3-}$ cluster is also presented.

The partial density of states (PDOS) of LN:Mg,Zr was calculated with the aforementioned optimal ${\mathrm{Mg}}_{\mathrm{Li}}^{+}$ + ${\mathrm{Zr}}_{\mathrm{Nb}}^{-}$ model, as shown in Figure 4a. Moreover, according to the 3${\mathrm{Mg}}_{\mathrm{Li}}^{+}$ + ${\mathrm{Mg}}_{\mathrm{Nb}}^{3-}$ defect model [26], the calculated PDOS of LN:Mg is described in Figure 4b for comparison. The PDOS clearly showed the DOS of each atom in the LN:Mg,Zr crystals, and the highest occupied valence band exhibits mainly O-2p features, whereas the lowest unoccupied conduction band mainly consists of Nb-4d electrons, meaning that the band gap of LN is determined by the transition energy of an electron transition from O^2−^ 2p-state to Nb^5+^ 4d-state, consistent with the widely accepted results [28]. 

Moreover, it can also be seen that the density of states of O-2*p* and Nd-4*d* in LN:Mg,Zr exhibit a distinctly decreased DOS at valence band maximum (VBM) and conduction band minimum (CBM), respectively, which elucidates the reduction of charge carriers in crystals [29]. The enhancement of resistance against UV optical damage of LN:Zr is considered as the reduction of UV photorefractive centers such as O^2−/−^ near ${\mathrm{Zr}}_{\mathrm{Nb}}^{-}$ [11,30]. It knows by the calculated DOS that the charge density of O-2*p* of LN:Mg is reduced dramatically by ZrO_2_ co-doping, which signifies that the UV photorefractive center O^2−/−^ of LN:Mg,Zr is sharply decreased. Moreover, the band gap of LN:Mg,Zr is calculated to be about 3.116 eV, which is significantly greater than that of LN:Mg (2.942 eV), and the increase in band gap of LN:Mg,Zr makes charge carriers excitation from O^2−/−^ more difficult, which powerfully proves the above analysis and further confirms the theoretical calculation corresponding to the experiment results. Based on the analyses above, we state that the reduction in the number of UV photorefractive canters O^2−/−^ and the increased band gap together result in the high resistance against UV optical damage of LN:Mg,Zr.

## 4. Conclusions

In summary, we have investigated the resistance against ultraviolet damage and electronic structures of LN:Mg co-doping with ZrO_2_ by experiment and first-principles calculations. It is found that ZrO_2_ co-doping greatly improves the ultraviolet damage resistance of LN:Mg. Meantime, first-principles calculations elucidate that the enhancement of resistance against UV damage of LN:Mg by co-doping ZrO_2_ is caused by both the increased band gap and the reduced ultraviolet photorefractive center O^2−/−^. This knowledge may open avenues to expand the nonlinear optical applications of LN:Mg to the UV region, and it can be an important candidate material for UV frequency conversion.

## Figures and Tables

**Table 1:** Formation energies of defect clusters in LN:Mg,Zr.

Defect Clusters	${\mathrm{Zr}}_{\mathrm{Li}}^{3+}$ $+{4V}_{\mathrm{Li}}^{-}$ $+{\mathrm{Mg}}_{\mathrm{Li}}^{+}$	${\mathrm{Mg}}_{\mathrm{Li}}^{+}$ $+{\mathrm{Zr}}_{\mathrm{Nb}}^{-}$	${\mathrm{Zr}}_{\mathrm{Nb}}^{-}$ $+4{\mathrm{Mg}}_{\mathrm{Li}}^{+}$ $+{\mathrm{Mg}}_{\mathrm{Nb}}^{3-}$
Formation energy (eV)	12.856	1.064	4.613

## Data Availability

The data presented in this study are available from the corresponding author on reasonable request.
